# Single molecule nanopore counting assay targeting small extracellular vesicle cargo for non-invasive monitoring of cerebral organoid development and health

**DOI:** 10.1038/s41598-025-31284-8

**Published:** 2025-12-12

**Authors:** S. M. Saiduzzaman, Ruiting Xu, Mohammad Julker Neyen Sampad, Ryan N. Hoffman, Spencer T. Seiler, Quinton Brail, Viktor Yurevych, Zachary J. Walker, Tanner N. Wells, Ephraim M. Ong, Thomas D. Yuzvinsky, Aaron R. Hawkins, Sofie R. Salama, Mircea Teodorescu, David Haussler, Holger Schmidt

**Affiliations:** 1 School of Engineering, University of California, Santa Cruz, CA 95064 USA; 2 Department of Molecular, Cell, & Developmental Biology, University of California, Santa Cruz, CA USA; 3 Department of Biomolecular Engineering, University of California, Santa Cruz, CA USA; 4 Electrical and Computer Engineering Department, Brigham Young University, Provo, UT 84602 USA

**Keywords:** Biotechnology, Nanobiotechnology, Biosensors, Engineering, Biomedical engineering

## Abstract

Organoids are three-dimensional tissue cultures intended to replicate in vivo organs such that their function can be analyzed for applications in drug discovery, diagnostics, and research. This requires the ability to assess organoid health, development, and function on the cellular and molecular level, possibly frequently and over long periods of time. Here, we report an assay for monitoring organoid development and health by tracking the molecular cargo of small extracellular vesicles (sEV) with an integrated nanopore sensor chip. Specifically, we implement amplification-free and label-free quantification of the organoid stress marker ENO1 produced by cerebral organoid tissue. We demonstrate that mRNA levels measured non-invasively in sEVs are representative of the amounts measured by PCR measurements of the tissue cells. We also quantify the ENO1 RNA load in sEVs over the course of 15 weeks and show that ENO1 expression levels are correlated with other physiological parameters such as organoid glucose consumption. These results illustrate the capability of single molecule nanopore sensors for providing simple, continuous, quantitative assessment of organoids’ phenotypes on the molecular level. This approach can be expanded to other molecular biomarkers such as protein transcripts, multiplexed analysis, and fully integrated in-line analysis in an automated tissue culture platform.

**Supplementary Information:**

The online version contains supplementary material available at 10.1038/s41598-025-31284-8.

## Introduction

The development and study of 3D tissue cultures (organoids) that mimic certain aspects of organ biology in vitro has emerged as a rapidly growing research field. Organoids can serve as simplified and accessible “minimal systems” to analyze the distinct contributions of various tissue components to complex morphogenetic processes with numerous potential applications^1^. Genetic modification of organoids enables disease modeling in a setting that closely resembles the physiological environment. Moreover, organoids can be efficiently grown as models of patient-derived healthy and diseased tissues, offering potential for patient-specific drug testing and the development of personalized treatment strategies^2–5^. Recent advances in pluripotent stem cells (PSC) and laboratory protocols have made it possible to generate 3D brain, gut, liver and breast models mimicking key functionalities of their counterpart human primary tissues better than their 2D cell culture models^6^. While these self-assembled 3D models are increasingly resembling primary tissues, challenges remain due to the inability to replicate the intricate complexities of tissue environments in the human body within a laboratory setting. For tissues representing a specific brain area, for example, it is challenging to grow organoids in the lab that contain all functionalities and cell type heterogeneities for that specific brain area. A major challenge in lab grown human brain organoids is the lack of distinct cellular subtype identity. Bhaduri et al.^7^ showed that cellular stress imposed by their in vitro cell culture conditions plays a crucial role in impairing cell type specifications in cerebral organoids. While cellular stress can be alleviated if the cortical organoid is transplanted into a mouse cortex, such an in vivo growth strategy faces limitations in scalability and experimental accessibility of the organoid tissue. It has been sufficiently difficult to solve the problem of cellular stress and other organoid defects in vitro that Vértesy et al.^8^ proposed an algorithm, Gruffi, which uses granular functional filtering to identify and remove data from stressed cells in an organoid scRNAseq dataset to better match data from primary tissue. The real solution will be better organoids. Organoid growth involves a months-long, complex process of initial cell aggregation, proliferation, migration and differentiation. To assess and confirm successful organoid genesis, it is critical to determine at all stages whether the organoids contain the desired cell types, and the degree to which the organoids accurately mimic the functions of the corresponding primary tissue^9^. Therefore, highly sensitive, non-invasive, and simple multimodal approaches for repeated measurement of molecular biomarkers are needed to monitor the organoid growth process. If results from these can be obtained in near real time, e.g. hours instead of days, then they also provide an opportunity to intervene when organoid health is compromised, e.g. when metabolic conditions deteriorate, or stress biomarkers are overexpressed. Seiler et al.^10^ have recently introduced a modular automated microfluidic cell culture platform that allows for continuous monitoring and control of organoid status via a highly automated, remotely controlled system. Programmable microfluidics and remote Internet of Things (IoT) access were implemented to monitor the macroscopic state of a cerebral organoid culture and enable scheduled exchanges of the culture media. Subsequent analysis of the cultures revealed that automation helped reduce glycolytic stress in the cell tissue. This platform serves as the basis for this work, which focuses on continuous, quantitative analysis of the molecular biomarkers produced by developing organoids.

There are several challenges to analyzing organoids and their molecular products. One issue is access to molecular biomarker products without sacrificing the tissue itself. Small extracellular vesicles (sEVs) (~ 50–150 nm) that are continuously being released by the live tissue hold great promise as proxy structures that can be obtained easily and in a non-destructive manner. sEVs are produced by numerous cell types and serve as a crucial pathway for intercellular communication^11–13^. They also transport various cellular components, including proteins, mRNAs, miRNAs, DNA, and lipids from cell to cell, influencing numerous physiological processes^14^. Several studies have reported the growing diagnostic^14–16^ and therapeutic^17–19^ potentials of sEVs. Brain organoids also shed sEVs that contain important cellular and organoid state information^20^, including full-length mRNA as well as miRNAs^21^. Therefore, monitoring sEVs released by cerebral organoids into the surrounding growth media can provide important insights into organoid growth and overall health, without the need to sacrifice tissue.

The second major challenge is to identify a suitable analysis approach that can quantify molecular biomarker content of sEVs accurately, quickly, and with limited complexity to make long-term longitudinal studies practical and affordable. The current gold standard methods are amplification-based nucleic acid detection (PCR, qPCR, RT-qPCR, LAMP), nucleic acid sequencing, and enzyme-linked immunosorbent assay (ELISA) analysis of proteins^9^. Genomic sequencing is highly sensitive, but involves highly complex sample preparation, library preparation, inherently complex sequencing processes, and significant data post-processing. In concert, these requirements make sequencing ill-suited for live and quick monitoring of relevant targets. qPCR is the gold standard for diagnostics due to its high specificity and sensitivity^22,23^. However, the need for thermal cycling, a reverse transcription step for RNA detection, problems with the amplification process, and the need for a standard calibration curve for quantitative cargo load analysis make qPCR a complex process^24^. Likewise, digital PCR (dPCR) is another quantitative tool for nucleic acids that provides excellent sensitivity, but relatively high complexity^25^. These methods are limited to analyzing nucleic acids. Proteins can be analyzed with ELISA and Western Blotting with relatively low sensitivity^9^ or by mass spectrometry, which is again more complex and costly.

Here, we introduce direct single molecule detection with integrated electrical nanopore sensors as a new approach to address the challenges of long-term organoid monitoring. Nanopore-based single molecule detection relies on changes in ionic current caused by nanoparticles crossing an insulating membrane with a nanoscale opening. This technology, using protein nanopores, is the backbone of commercial next-generation DNA sequencing technology^26^. However, the principle can also be used to detect other biomolecules, including DNA^27,28^, RNA^29^, proteins^30,31^, and small molecules^32^, using cheaper and more stable solid-state nanopores.

Recently, we have introduced a chip-based solid-state nanopore sensor that is based on trapping microbeads that carry molecular targets at the location of a nanopore. When the targets are (thermally) released from the beads they are rapidly drawn through the nanopore and counted^33^. This trapping-based target enrichment at the nanopore enables label-free, amplification-free direct quantification at clinically relevant concentrations at the femtomolar and attomolar level and makes this approach ideal for monitoring sEVs. In this study, we use a nanopore sensor assay to monitor the mRNA content of the metabolic stress gene ENO1 over the course of a 15-week human cerebral organoid growth study involving two different growth media. We demonstrate that this approach correctly reflects the ENO1 levels in the cell tissue, identifies the dependence of ENO1 expression on the growth media, and correlates the ENO1 expression in sEVs with physiological parameters. These results establish nanopore sensors as an ideal molecular diagnostic companion for organoid research with the potential for future expansion to a multitude of targets and full system integration.

## Results

### 3D tissue culture platform for long-term organoid studies

**Fig. 1:**
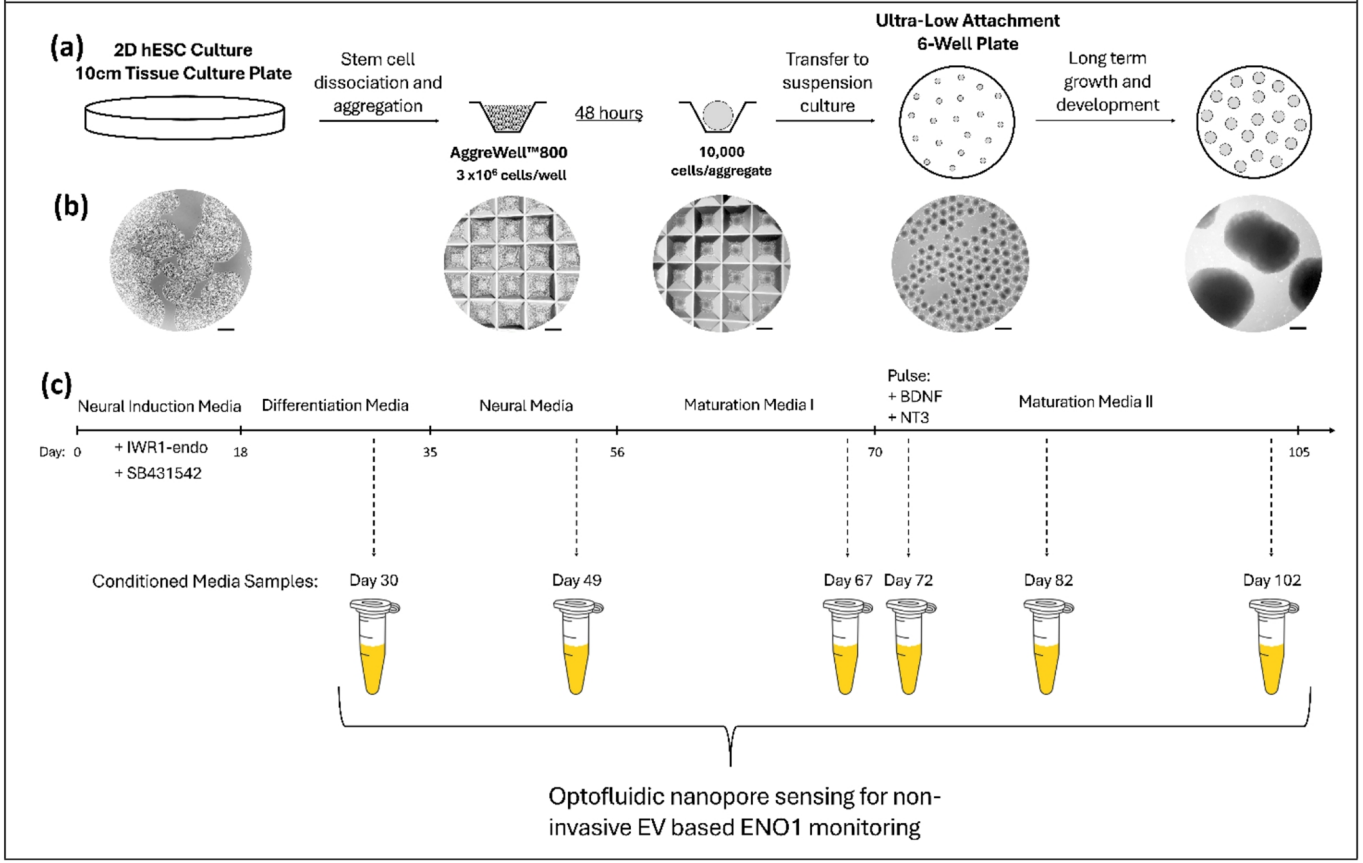
Experimental study design: (**a**) A schematic overview for the generation and culturing of cerebral organoids. Human embryonic stem cells (hESCs) are grown on 10 cm tissue culturing plates. Once 70–80% confluent, hESC are dissociated into single cell suspension and plated into Aggrewell™800 wells at 3 million cells per well. Plates are spun to aggregate stem cells and given 48 h to adhere into stem cell aggregates. Stem cell aggregates are then transferred to ultra-low attachment 6-well plates. These aggregates are then grown in 4 ml of suspension media and patterned toward cerebral specificity for 105 days. (**b** )Brightfield imaging showing corresponding stages of the organoid generation process, including 2D stem cell culture, day 0 aggregation, day 2 stem cell aggregates in Aggrewell™800, day 2 stem cell aggregates transferred to suspension culture, and day 56 cerebral organoids in suspension culture. Scale bars are equal to 500 μm. (**c**) A schematic of the timeline and specific media and patterning regime used to develop cerebral organoids. Conditioned media samples were harvested at six timepoints for optofluidic nanopore sensing for non-invasive EV based ENO1 monitoring.

Long-term studies of cerebral organoids are of great interest and importance as they have the potential to shed light on different stages of neuronal development, starting from the earliest stages of tissue growth. Previously, we developed automated tissue culture platforms that integrate microfluidic media handling, electrophysiology measurements and imaging in a unified system and can control and analyze organoids over many days^10,34^. We used the system shown in Fig. 1 to explore the impact of the physiological parameters present in the organoid growth environment on long-term stress and metabolic levels.

We conducted a 15 week (105 days) long study to follow the development of cerebral organoids through different developmental stages under different growth conditions. Stem-cell-derived cerebral organoids differ from primary cerebral cortex tissue, most notably in upregulating glycolysis and endoplasmic reticulum stress while downregulating oxidative phosphorylation^7,35^. Downstream issues of cell type fidelity and differentiation in organoids are correlated with these metabolic differences. Non-physiological and highly variable cell culture media analyte concentrations (pH, glucose, lactate) may degrade the cerebral organoid model. Therefore, the comparison between growth media that are maintained at high glucose levels present in standard cell culture media (henceforth “High Glucose Media”) and media whose glucose level was continuously adjusted to stay within physiological limits (“Adaptive Glucose Media”) is of particular interest.

The state of the culture was monitored using bulk measurements of the culture media supernatant with a Vi-CELL MetaFLEX instrument (pH, pO_2_, pCO_2_, glucose, lactate, and electrolytes). The number of organoids per well was controlled at 20 organoids per well from day 18 until day 70 and 10 organoids per well from day 70 to day 105. Details of the organoid growth protocol and system setup are included in the "Materials and methods" section. While the development of organoid cultures can be monitored on the molecular level, e.g. to track gene expression profiles, techniques that provide single molecule sensitivity, such as fluorescence imaging, sequencing, PCR and others, are generally complex and/or destructive to the tissue, hence not suited for automated application at frequent intervals over long periods of time as discussed above. Therefore, we extracted the conditioned media from both growth conditions over the course of the experiment (Day 30, 49, 67, 72, 82, 102) to measure the concentration of the ENO1 stress gene in sEVs using an integrated nanopore sensor and with three specific goals: (1) to show that sEVs can serve as an accurate, non-destructive proxy for the ENO1 levels in the actual cell culture; (2) to show that the concentration can be tracked over long periods of time with a low complexity, potentially automatable assay, and (3) that the results correlate with other metabolic measurements, creating the potential of using a single measurement to assess multiple aspects of the homeostatic state of the organoid culture.

**Fig. 2:**
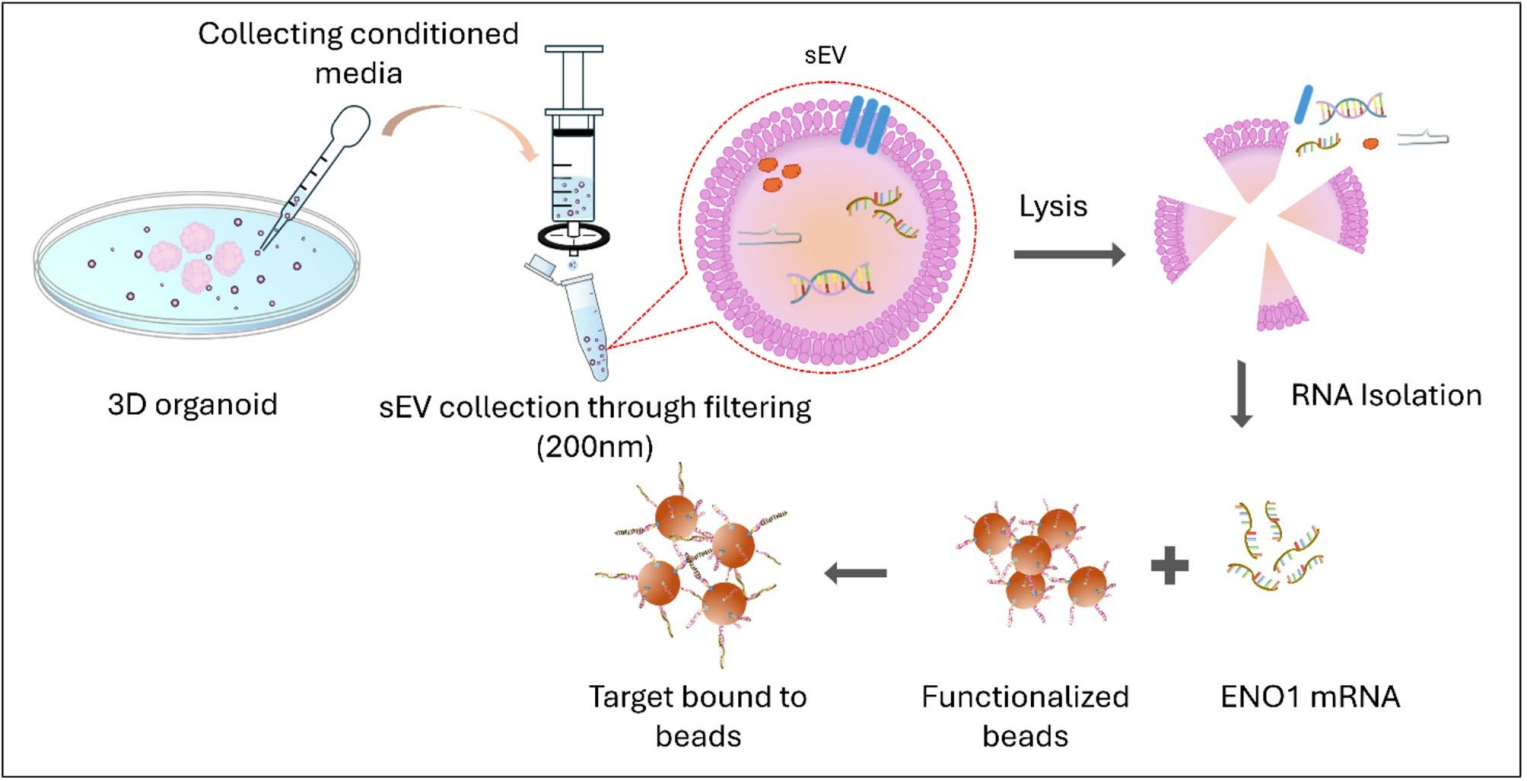
ENO1 mRNA isolation from sEVs derived from cerebral organoids followed by capture on microbeads for single molecule solid state nanopore sensing.

ENO1 is a metabolic biomarker^36^ that translates into alpha enolase-1, a major enzyme in the glycolytic pathway. It is significantly associated with the glycolytic stress subpathway, along with other metabolic genes^7,8^. Other work has shown that in a quantitative comparison of iPSC-derived motor neurons and their secreted EVs, ENO1 is found to be more abundant in EVs than cells^37^. Figure 2 illustrates the sample preparation steps for isolating ENO1 from organoid-derived sEVs with high specificity for integrated optofluidic nanopore sensing. The sEVs are collected from the organoid growth media, lysed, and mixed with microbeads whose surface is functionalized with an ENO1 specific pulldown sequence. Details of the oligo pulldown design and protocol are provided in the "Materials and methods" section.

### Single molecule nanopore sensor

Solid state nanopore sensing relies on connecting the two sides of a thin, non-permeable, and electrically insulating membrane through a nanoscale orifice^38^. Both sides of the membrane contain ionic liquid. An electrical bias voltage across the two sides of the membrane creates a hemispherical electric field that is concentrated within a radius of a few micrometers around the pore. An analyte has to be located within this capture radius in order to be pulled through the nanopore^39,40^, which limits the applicability of the nanopore sensing at low concentrations. Recently, we have developed an integrated optofluidics-based Trap Assisted Capture Rate Enhancement (TACRE) method, which concentrates targets captured on microbeads at the nanopore. Upon release from the beads, the molecules are rapidly and efficiently drawn through the nanopore, creating a characteristic current blockade signal^41^. The TACRE method can enhance the nanopore sensing rate over 2000 ×^42^ and was shown to deliver qPCR level performance for label-free, amplification-free, reference-free, and quantitative measurements of viral RNAs from a variety of biological fluids^33^.

**Fig. 3:**
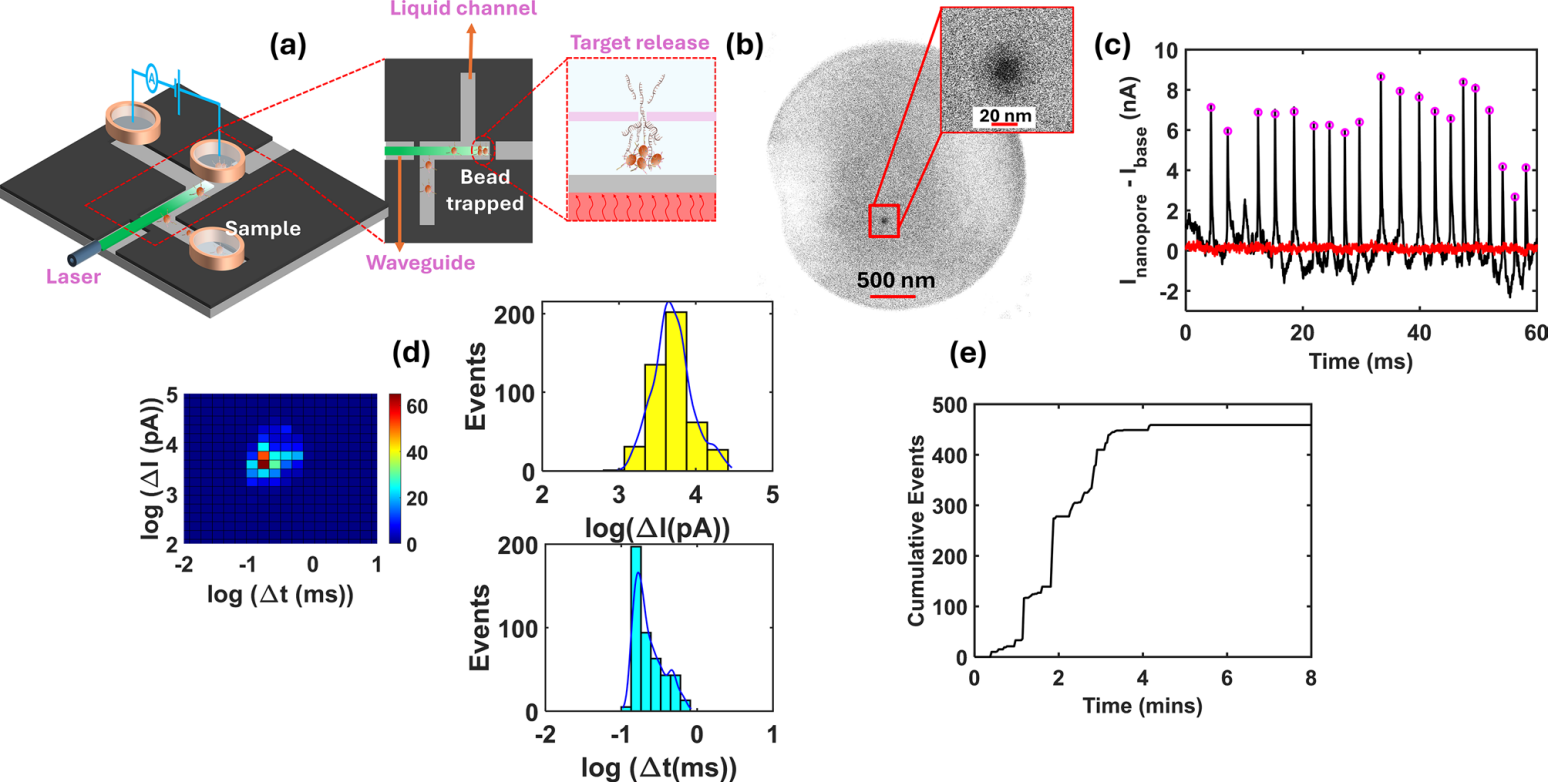
Solid state nanopore sensing based sEV ENO1 quantification from cerebral organoid growth media: (**a**) Device schematic illustrating optofluidic trapping of ENO1 enriched microbeads; release & capture of ENO1 mRNA targets (**b**) SEM image of 20 nm pore right above the trapping area (**c**) Translocation (pink) events detected in the ionic current trace (black) & a corresponding negative (red) control current trace (**d**) Heat map of signal depth(ΔI) and dwell time (Δt) of all translocation events detected in nanopore (**e**) Translocation event distributed over time

Figure 3a shows a schematic view of the optofluidic nanopore chip and the experimental setup employed for ENO1 quantification from sEVs using the TACRE principle. The device consists of a microfluidic channel with a cross-section of 10 μm × 14 μm on a 12 mm × 5 mm silicon chip. This microfluidic channel is defined through selective etching and a silicon dioxide (SiO_2_) deposition process. At the center of the device, the microfluidic channel intersects with a solid-core (SC) waveguide measuring 10 μm × 6 μm, forming a 100 μm long horizontal optofluidic particle manipulation region. This horizontal section is extended by 20 μm to create a low-flow protrusion cavity designed to isolate particles from the main channel’s fluid flow. A 300 nm thin SiO_2_ membrane is selectively deposited around the central optofluidic region, encapsulating the microfluidic channel and providing a thin membrane structure that facilitates integration of a 20 nm wide nanopore using a focused Ga^+^ ion beam (see Fig. 3b). The detailed fabrication procedure of the chip is described in the "Materials and methods" section.

Fluidic and electrical access to the microfluidic channel is provided by three metallic reservoirs that are attached to the chip using wax. An electric potential difference is applied between the outlet and the nanopore reservoirs using Ag/Ag electrodes (see Fig. 3a). Ionic current signals across the nanopore are measured via a patch-clamp current amplifier (Axopatch 200B, Molecular Devices) connected to the electrodes.

A single-mode optical fiber is coupled to the solid-core waveguide on the other side of the protrusion region. The fiber is coupled to a 532 nm green laser. The laser beam (green in Fig. 3a) pushes the ENO1 mRNA-enriched microbeads to the low-flow protrusion cavity. When the chip is heated and an electrical potential is applied across the outlet reservoir and the nanopore reservoir, released ENO1 mRNA are translocated through the nanopore. The temperature is maintained at 50 °C for the first two and half minutes and then the heater is turned off.

Figure 3c shows a segment of the nanopore current signal that shows characteristic spikes caused by single ENO1 RNAs extracted from sEVs contained in the adaptive glucose organoid media on Day 30 of the study.

A negative control of the current signal in the presence of functionalized beads at the nanopore, but in the absence of targets is also shown (red line), indicating that the probes are strongly bound to the beads so as to not produce spurious false positive signals. The current signatures can be analyzed for their duration (Δt) and their depth (ΔI). The resulting distributions are shown in Fig. 3d, both individually and as a 2D heat map. The tightly clustered events indicate that the signals originate from a single particle type, here mRNA. Figure 3e shows the progression of the cumulative particle count, demonstrating that the targets are rapidly captured and counted within five minutes. The ENO1 concentration can then be determined from the detected number of nanopore translocations, the number of trapped beads, and the starting sample volume (0.5 mL) as described previously^33^. For instance, we find the concentration values of 1.4 × 10^6^/ml (adaptive media day 30) and 1.36 × 10^6^/ml (high glucose media day 30) with a statistical uncertainty determined by variations in the number of targets per bead due to the thermodynamic nature of the binding process. Assuming a binomial distribution, the variance *s* of the number of targets per bead is

1 $$\sigma \left({n}_{t}\right)={n}_{t}\times\:\frac{{N}_{mRNA}}{{n}_{tot}}\times\:\left(1-\frac{{n}_{t}}{{n}_{tot}}\right)$$

Where *n*_*t*_ is the number of trapped beads, *n*_*tot*_ is the total number of beads mixed with the sample, and *N*_*mRNA*_ is the number of RNA molecules detected with the nanopore sensor. We find that the error amounts to 4.6% and 4.1%, respectively.

## mRNA measurements from sEVs are indicative of the content in cell culture

**Fig. 4:**
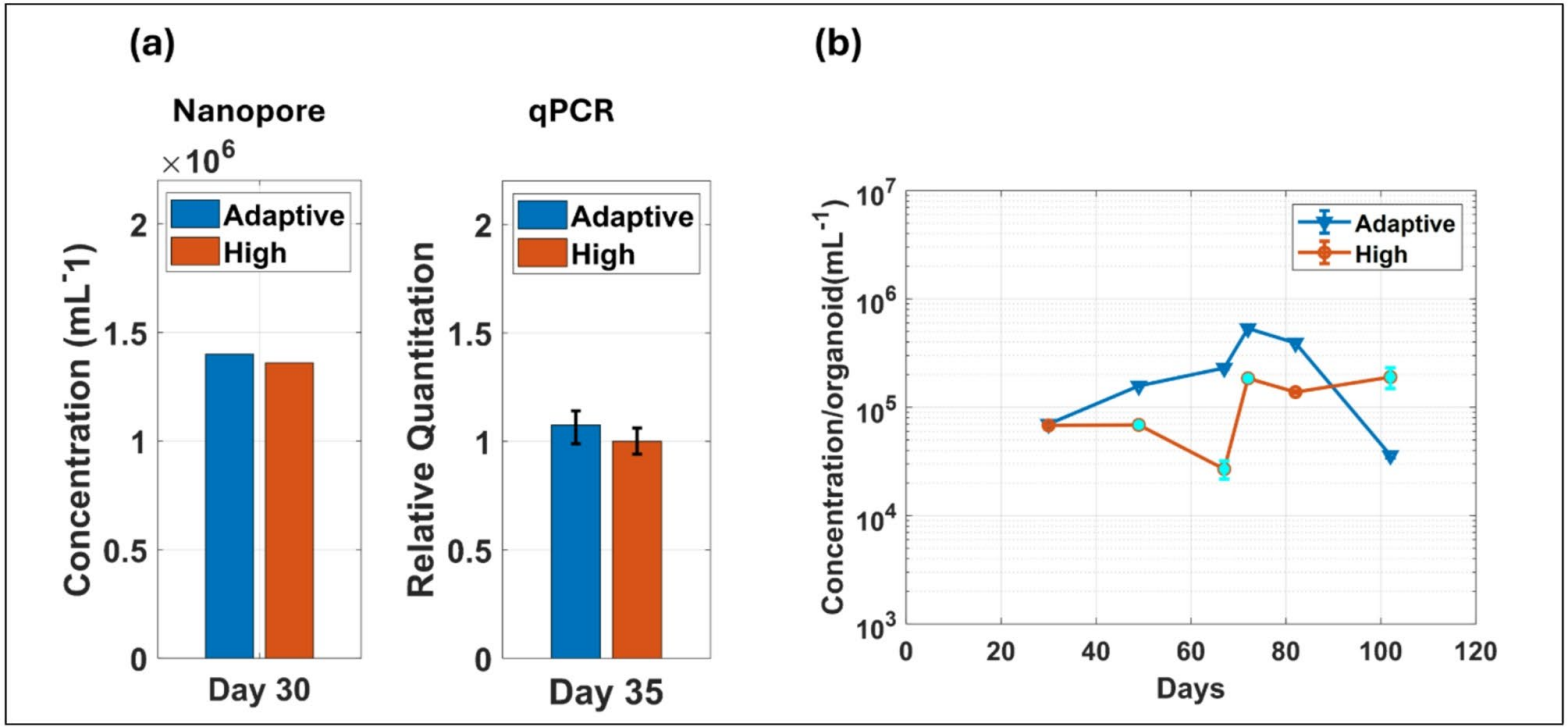
Long-term ENO1 monitoring from cerebral organoid-derived EVs: (**a**) Direct EV ENO1 count from nanopore (left) on day 30 vs. qPCR of ENO1 expressed in organoid cells (right) on day 35 for both media (**b**) EV ENO1 concentration per organoid over 15 weeks organoid growth period in two different conditioned media – Adaptive & High Glucose.

Our first goal is to establish the suitability of measuring the transcript number in EVs as a proxy for the target level in the cell culture. To this end, we carried out a TaqMan qPCR assay on organoid tissue cells to assess the ENO1 levels for both adaptive and high glucose media. (Details are described in the "Materials and methods" section.) We then ran the simpler, quantitative nanopore assay using the conditioned media supernatant around the same point during the organoid development timeline. The results for both approaches are displayed in Fig. 4a. The qPCR error bars were calculated for 95% confidence interval of relative quantification as generated by QuantStudio™ v1.7.2. Both assays show that ENO1 is expressed in similar quantities in both types of media, with the level in the adaptive media being slightly higher. This demonstrates that direct detection of the ENO1 mRNA in sEVs reproduces the development in the cell culture. Note that the nanopore assay is both quantitative and reference-free and, therefore, yields more information than the qualitative, more complex and time-consuming qPCR approach.

## Long-term mRNA quantification from sEVs

Having established the utility of measuring ENO1 content in sEVs released into the culture media as a proxy for the presence of this metabolic marker in the cell culture, we used the nanopore assay to track the ENO1 level per organoid across the entire duration of the organoid study. The results are shown in Supplementary Table S1 and in Fig. 4b where the amount of sEV-derived ENO1 mRNA is plotted versus time.

First, we find that the ENO1 level for both growth conditions varies between 10^4^/ml and 10^6^/ml, i.e. in the attomolar range. This validates the use of target enhancement at the nanopore using trapped carrier microbeads. Second, error bars reflecting the standard deviation of the observed target number according to Eq. (1) are displayed in the figure but too small to be visible in the logarithmic scale, showing that the measurement uncertainty is negligible compared to the variation over the course of the organoid development. The cyan points in Fig. 4b provide the duplicate values with error bars for the respective days determined by the TACRE assay. These points demonstrate the reproducibility of the nanopore assay and establish confidence about the nanopore results for the other days for which no replicate measurements were taken due to sample volume scarcity. Third, we find that the ENO1 levels are initially comparable; however, the level drops in the adaptive media after day 70 while it continues to increase for the high glucose media. This suggests a reduction in organoid stress in the adaptive glucose culture after day 70 and can be investigated further with complementary measurements.

## Correlation of ENO1 biomarker with other organoid characteristics

The ability to track molecular biomarkers easily and repeatedly opens the door to correlating the molecular data, here the ENO1 expression levels, with other characterization techniques for the organoid culture, in particular in the context of a larger, automated platform.

**Fig. 5:**
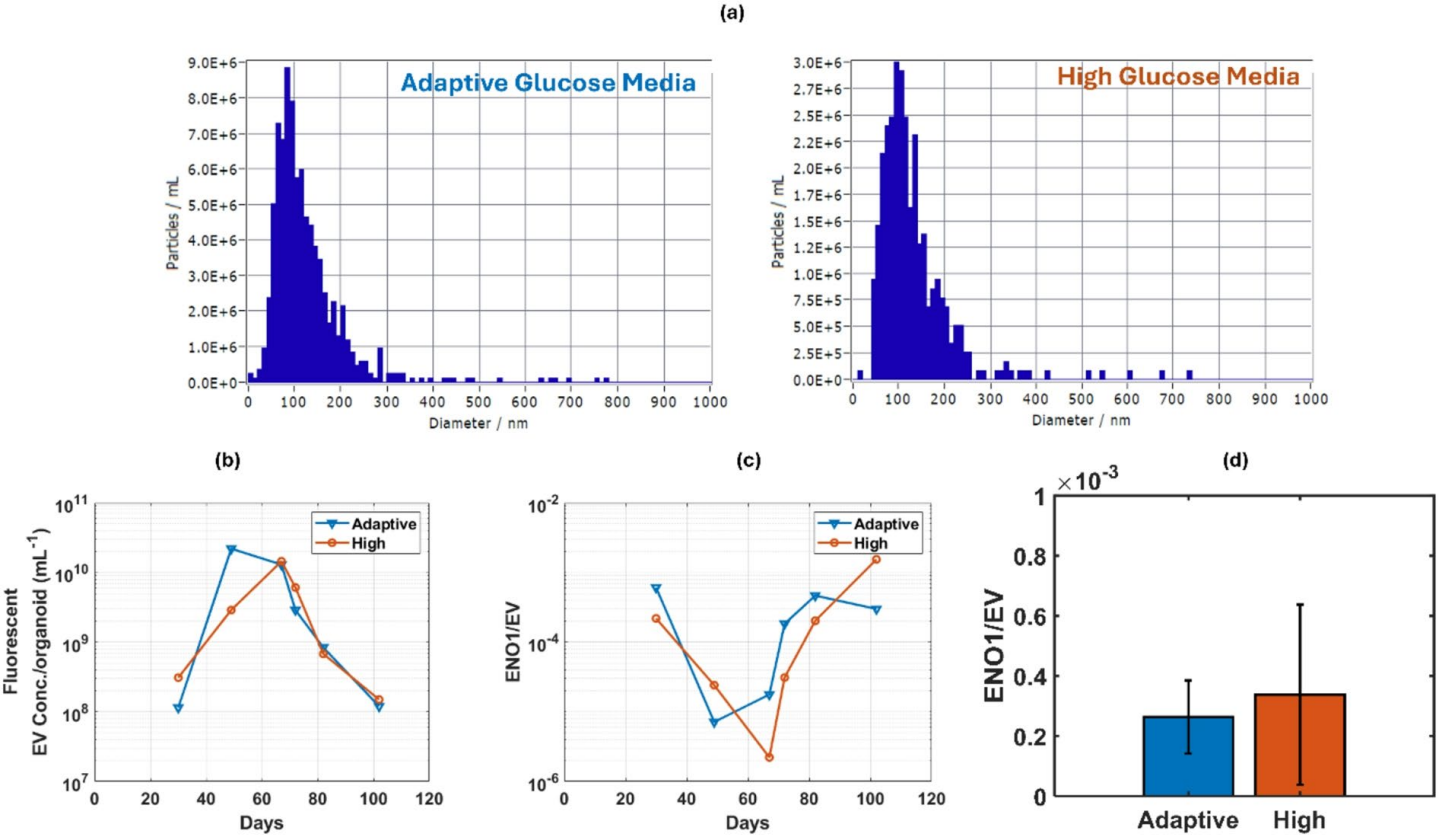
ENO1 packaging in Cerebral organoid EVs: (**a**) Size profile of sEVs from Day 67 adaptive & high glucose conditioned media and (**b**) Concentration of sEVs derived from cerebral organoid over different growth periods; (**c**) ENO1 packaging rate in EVs & (**d**) mean ENO1 packaging rate in sEVs over whole growth period.

As a first representative example, we evaluated the relation of the ENO1 concentration with their sEV source nanoparticles. A nanoparticle tracking analyzer (NTA –ZETAVIEW, Particle Metrix) was used to measure sEV concentration and size distribution for each sample. Figure 5a shows the example of the size distributions for the adaptive and high glucose media sample, respectively, taken during the neurogenesis stage of the cerebral organoids (Day 67) The data confirm the effectiveness of the 200 nm filter through which each sample was run and shows a median sEV size of 100 nm and a concentration of 1.30 × 10^10^/ml-organoid for adaptive glucose media. For the high glucose media, a median sEV size of 107 nm and a concentration of 1.45 × 10^10^/ml-organoid are observed. Taking NTA measurements for all samples allowed us to track the evolution of both the sEV concentration (Fig. 5b) and the number of ENO1 mRNAs per sEV (Fig. 5c). We see that the ENO1 packing ratio, i.e. the expected number of ENO1 mRNAs per sEV, is overall very low, especially when the sEV concentration is high (Day 50–70). This indicates that during this period the organoids produce a large number of extracellular vesicles, most of which do not contain the ENO1 stress marker. Overall, the results suggest that high metabolic demand during early differentiation might be related to the high ENO1/EV ratio we observe around day 35. This is consistent with ectopic activation of glycolysis pathways in early organoids reported by Bhaduri et al.^7^. Mulder et al.^43^ reported that majority of cell types develop between days 35–60 in some organoid models, which could contribute to a reduced ENO1/EV ratio around day 70 as seen in Fig. 5c through stabilized differentiation. At later stages, size and diffusion-limited hypoxia and necrosis might drive renewed stress, as noted by Lancaster et al.^44^, consistent with the post-day 70 increase in the ENO1/EV value. As shown in Fig. 5d, the average ENO1 packaging rate in sEVs over the whole growth period is 2.6 × 10^− 4^ with a 1.22 × 10^− 4^ standard deviation for adaptive glucose media and slightly higher at 3.37 × 10^− 4^ in the high glucose media, albeit with a larger 3 × 10^− 4^ standard deviation.

**Fig. 6:**
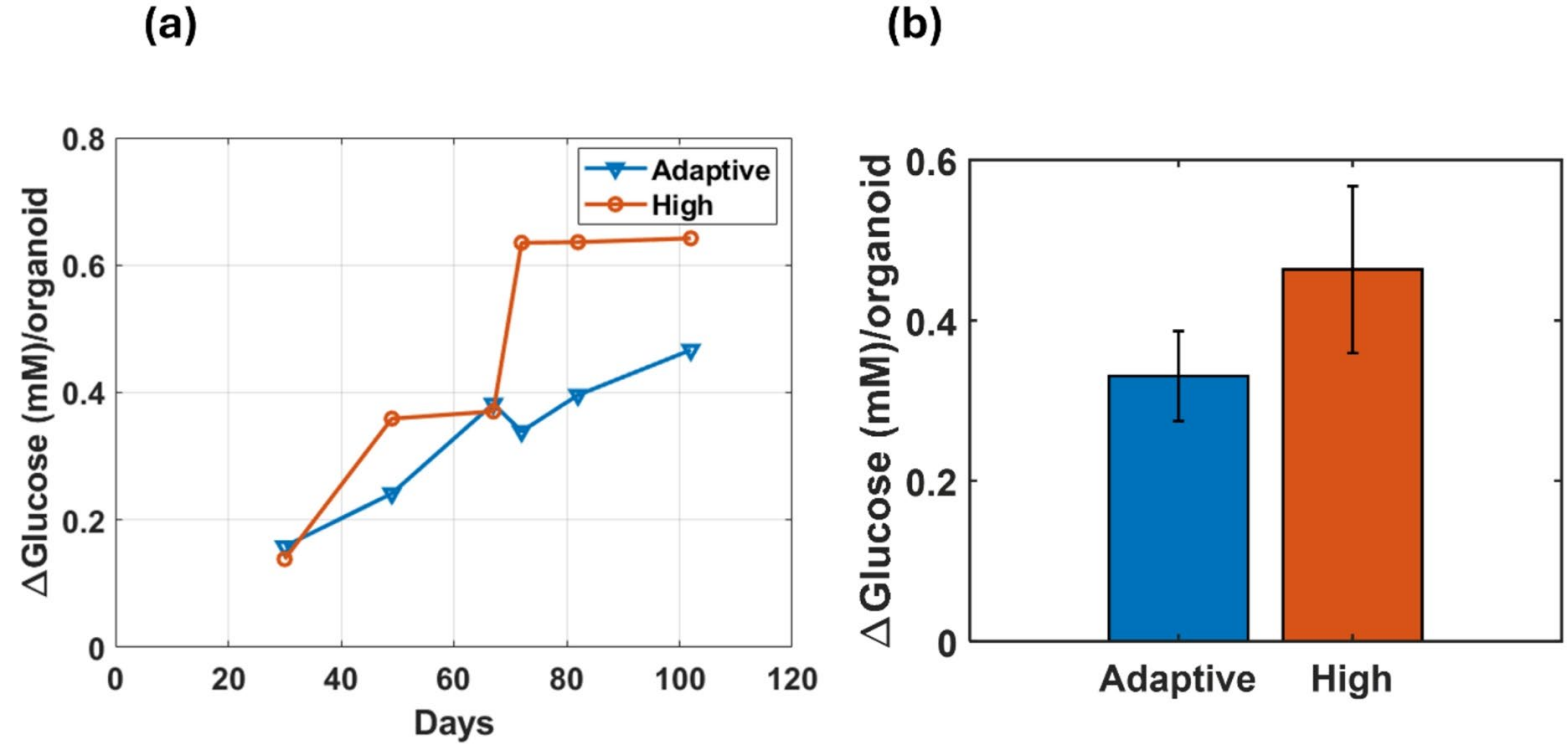
Glucose consumption in organoid media: (**a**) Glucose consumed by each organoid over whole growth period (**b**) mean glucose consumption over whole growth period.

Finally, we assessed the correlation between ENO1 packaging and glucose consumption in the organoid culture. To this end, the glucose level at the 0th hour and 48th hour following a media change were recorded by the Vi-CELL MetaFlex with 200 µL conditioned media. To calculate the glucose consumption/generation per organoid, the difference between the 0th hour and 48th hour was divided by the number of organoids in the well. Figure 6a displays the trend in the change of glucose over the course of the study and shows that organoid glucose consumption was better controlled in adaptive glucose media (blue) than the high glucose media (orange), in accordance with the goal to keep the organoids within physiological metabolic limits.

Figure 6b summarizes this behavior by displaying the average glucose consumption values over the course of the study, where the error bars indicate the standard variation. For the adaptive glucose media, we find an average glucose consumption of 0.33 mM/organoid with a 0.11 mM/organoid standard deviation while the values for the high glucose media are 0.46 mM/organoid with a 0.2 mM/organoid standard deviation, higher than for the adaptive media. Figure 6b shows the similar qualitative difference in standard deviation between the media as Fig. 5d, suggesting that changes in the glucose consumption may impact the ENO1 production and secretion from the organoid culture.

## Discussion

In this work, a system for rapid, non-destructive, and sensitive monitoring of molecular biomarkers generated by (cerebral) organoid cultures is presented. The system relies on solid state nanopore based sensing, where the local concentration enhancement of mRNA targets around the nanopore enables rapid, simple, and direct quantification. As such, our platform has sufficiently low complexity to enable frequent, repeated measurements on small extracellular vesicles as proxies for the state of the cell culture. To demonstrate the capabilities of this approach, the stress gene ENO1 was monitored over 15 weeks in two nutritionally different growth media. It was observed that only a small fraction of sEVs contain ENO1 mRNA, but with billions of sEVs collected per organoid, very accurate measurements of ENO1 concentration in sEVs are possible. We showed that ENO1 concentrations are in the attomolar range and could be successfully quantified and tracked by the nanopore chip. The ENO1 measurements were then correlated with complementary organoid characterization measurements, and possible correlations with glucose consumption were identified.

The integrated nanopore system is also extremely versatile and offers additional possibilities for a future expansion of capabilities. For example, since ENO1 mRNA packaging is a rare event, it is extremely important to analyze the sEVs at the single EV level. We have previously shown that nanopores can be electrically gated to deliver a user-determined number of nanoparticles into a fluidic channel for subsequent downstream analysis^32^. This approach can be applied to selecting individual sEVs of a particular size range for analysis of their molecular cargo.

Moreover, the nanopore sensor is target-agnostic, i.e. direct quantification using trapping-enhanced nanopore sensing is not limited to nucleic acids. By using carrier beads that are functionalized with antibodies, proteins can also be detected with single molecule sensitivity and at ultralow concentrations^45^. Since sEVs can contain proteins specific to neural cell types and sEV cargo content variation can correspond to cortical organoid growth stages^46^, protein analysis can provide important insights into cell type and neurodevelopmental stage for the cells within the organoid.

Rapid detection capability is a major feature of our nanopore-based quantification system. In this study, the total assay time including RNA extraction, sample incubation, and detection, was approximately 2 h. However, the target release and capture with nanopore step itself requires only ~ 5 min. For comparison, RT-Lamp based assays have been reported delivering results in 30 min. But additional sample preparation steps can take up to 40–60 minutes^47^. Note that none of the assay preparation steps were optimized for speed in this study. Previously, we have shown it is possible to optimize and reduce the assay time to be within 1 h while retaining the sensitivity in detection^48^.

Further advancement could be made through integrating on-chip sample preparation and delivery steps with the nanopore detection module^49–51^. This would lower the sample volume requirement and enable full integration with automated organoid tissue culture systems^10^. In conjunction with multiplexed target detection, such integrated systems could essentially measure the organoid’s molecular phenotype in real time. For instance, monitoring four glycolytic enzymes^10^- ALDOA, ENO1, HK1, and PGK1 would give an even more accurate picture of glycolytic stress, and given this information in real time would allow an automated system to take preventive measures such as glucose level control to modulate the glycolytic enzyme expression in organoids. Moreover, incorporating engineered cerebral organoids^52,53^, sliced organoids^54^, or air–liquid interface (ALI) organoids^55^ as control groups with resolved cell stress would strengthen these non-invasive EV based measurements, providing baselines for comparison and helping to distinguish glucose-induced transcriptomic expression changes in the glycolytic pathway from baseline organoid stress. This level of transcriptomic target multiplexing can be implemented in our system in various ways, e.g. by designing carrier beads with different release temperatures for each target, or by directing different carrier beads towards different nanopores in a multi-channel architecture.

In conclusion, this work establishes that an integrated optofluidic nanopore system can monitor cerebral organoid released sEV cargo information and thereby provide novel insights about the organoid’s composition and state non-invasively. In the future, these systems could enable continuous or on-demand, non-invasive monitoring at the single or multiplexed molecular biomarker level, providing real time information that would allow an automated controller to grow organoids better mimicking their primary tissue counterparts.

## Materials and methods

### Optofluidic nanopore device fabrication

Optofluidic nanopore devices were fabricated on flat 100 mm diameter silicon wafer^56^. A 3 mm long fluidic channel was defined by anisotropic etching into the silicon substrate using an STS ICP Multiplex ASE reactive ion etch (RIE) tool. The microfluidic channel mask consists of multiple inlets leading to a single channel. A section of the channel contains a 100 μm long optical trapping region that interfaces with a solid core waveguide and a 20 μm protrusion cavity isolating optically trapped beads from the active fluid flow. The etched channel cross section is 10 μm × 14 μm. To ensure optical mode alignment between the liquid channel and solid core waveguide, a pedestal was etched with an RIE for intersecting solid-core (SC) waveguides. Thus, the waveguides are 3 μm lower than the top of the liquid channel. Additionally, the second etch defines the liquid channel wall thickness to approximately 2.5 μm. The channel wall was thermally oxidized to convert silicon into silicon dioxide (refractive index *n* = 1.44) by heating the wafer in a furnace at 1,100 °C for 10.5 h, forming the bottom, low-index cladding for the solid-core (SC) waveguides. A 3 μm thick, high-index (*n* = 1.51) silicon dioxide layer was then deposited over the wafer using plasma-enhanced chemical vapor deposition (PECVD), and a 10 μm wide waveguide intersecting the optofluidic channel was defined by photolithography and reactive ion etching (RIE). To cover the microfluidic channel, a 300 nm thin PECVD silicon dioxide (*n* = 1.51) membrane was grown over the entire wafer after the channels were first filled with a sacrificial SU8-2000.5 polymer through capillary action ( with the polymer’s naturally formed meniscus shaping and supporting the membrane^57^. A 2 μm thick low-index (*n* = 1.46) silicon dioxide layer was then deposited over the channel, excluding the central trapping region, to provide mechanical strength and additional cladding for the SC waveguide. Finally, the sacrificial polymer was removed by chemical etching with sulfuric acid and hydrogen peroxide, creating a hollow channel with a 300 nm thin suspended silicon dioxide membrane in the optofluidic region, facilitating the integration of a solid-state nanopore sensor. The integrity of this thin oxide membrane was confirmed through electronic voltage-current measurements before nanopore milling.

An FEI Quanta 3D FEG Dual beam system with a gallium ion beam operating at 30 kV and 10 pA was used to mill a nanopore into the 300 nm thick silicon dioxide membrane atop the protrusion cavity. The nanopore fabrication process begins by milling a 1 μm diameter circular microwell into the membrane, creating a locally thinner region. A ~ 20 nm nanopore is then drilled through the remaining thinner oxide using a 20 ms dwell time at a single point, controlled by the Nanometer Pattern Generation System software (JC Nabity Lithography Systems). If the initial nanopore size exceeds the desired dimensions or if any visible cracks appear in the thinner oxide membrane, an additional tetraethyl orthosilicate-based nanopore shrinking step is applied. Throughout both the microwell and nanopore fabrication processes, monitoring is performed using a scanning electron beam operating at 5 kV and 6.7 pA.

## Organoid generation and culturing

Human embryonic stem cells (H9) are grown two-dimensionally on tissue culturing plates using StemFlex media until plates are 70–80% confluent. 2D stem cells are dissociated to single cell suspension with Accutase and plated into an Aggrewell™800 aggregation well at 3 × 10^6^ cells/well in AggreWell™ Media, which results in 10,000 cells per aggregate. On the day of aggregation, media is supplemented with 10 µM of Y-27,632. On day 2, stem cell aggregates are transferred to wells of an ultra-low attachment 6-well plate containing 4 ml of media. Stem cell aggregates are patterned from day 1 to day 18 with 3µM IWR-1-endo and 10µM SB431542 supplemented into AggreWell™ Media to guide development towards cerebral specificity. Cerebral organoids are provided differentiation media from day 18 to 35, which is composed of DMEM/F12 supplemented with 1% N2, 1% GlutaMax, 1% lipid concentrate, 1% penicillin-streptomycin, and 1% amphotericin B. Cerebral organoids are provided neural media from day 35 to 56, which is composed of DMEM/F12 supplemented with 10% ESC qualified FBS, 1% N2, 0.01% heparin, 1% GlutaMax, 1% lipid concentrate, 1% penicillin-streptomycin, 1% amphotericin B, and 0.12 mg/ml Geltrex. Cerebral organoids are provided maturation I media from day 56 to 70, which is composed of DMEM/F12 supplemented with 10% ESC qualified FBS, 1% N2, 2% B27 without vitamin A, 0.01% heparin, 1% GlutaMax, 1% lipid concentrate, 1% penicillin-streptomycin, 1% amphotericin B, and 0.12 mg/ml Geltrex. Cerebral organoids are provided maturation II media from day 70 to 105, which is composed of DMEM/F12 supplemented with 10% ESC qualified FBS, 1% N2, 2% B27, 0.01% heparin, 1% GlutaMax, 1% lipid concentrate, 1% penicillin-streptomycin, 1% amphotericin B, and 0.12 mg/ml Geltrex. Conditioned media samples were harvested from culture on day 30, 49, 67, 72, 82, and 102 and provided for optofluidic nanopore sensing for non-invasive EV based ENO1 monitoring. On day 70, the organoid density per culturing well was split from 20 organoids/well to 10 organoids/well. From day 70 to day 76, cultures were provided a maturation patterning pulse of 10µM BDNF and 10µ NT3.

### **Adaptive glucose culturing**

The adaptive glucose culturing condition began on day 18 of cerebral organoid culturing. Glucose depleted DMEM/F12 base media was used for differentiation media and Brainphys™ media was used as the base media for neural media and maturation media I/II formulation for this condition. Glucose was then supplemented into the media for each 48-hour media exchange to provide sufficient glucose for the next 48-hour feed cycle, while maintaining this adaptive glucose concentration range centered at 3.6mM glucose to represent physiological glucose concentration. Media samples were analyzed for metabolites prior to and immediately after each media exchange to calculate the glucose consumption rate of each well over the 48-hour feed cycle. This glucose consumption rate was used to provide a specific concentration of glucose to be supplemented for the next 48-hour feed cycle.

### Organoid cell-RNA quantification using qPCR

Organoids were dissociated in 500 µL QIAzol™ Lysis Reagent at day 35 and RNA was extracted via ethanol precipitation and column purification. cDNA was prepared via Transcriptor First Strand cDNA Synthesis Kit using 500ng of RNA. Real time qPCR was conducted on a Applied Biosystems^®^ QuantStudio™ 6 Flex Real-Time PCR System using Kappa PROBE FAST qPCR Master Mix with Integrated DNA Technology predesigned probe assays targeting ENO1 and housekeeping gene B2M. ENO1 relative expression was calculated via 2^–∆∆Ct^ method normalized to high glucose condition.

B2M probe: 5-′/56-FAM/CCTGCCGTG/ZEN/TGAACCATGTGACT/3IABKFQ/-3′.

primer 1: 5-G′ GACTGGTCTTTCTATCTCTTGT-3′.

primer 2: 5-A′ CCTCCATGATGCTGCTAC-3′.

ENO1 Probe: 5′-/56-FAM/CGAGACCA/ZEN/GTGGCTAGAAGTTCAC/3IAbkFQ/-3′.

primer 1: 5-G′ CTTTACTTCACCTCGGT-3′.

primer 2: 5-TGGCATGGATCTGAGAATAGAC-3′.

the FAM is a dye on the 5′ end of the probe, and the ZEN as well as the 3IABkFQ are quenchers on the probe.

### sEV collection and characterization

The collected conditioned media was centrifuged at 2000 rpm for 10 min to get rid of the cell debris and filtered with a 200 nm Millex^®^ Syringe-driven Filter Unit to collect the sEVs. Next, the 10x SYBR Gold solution was prepared in 1 × TE buffer and filtered through a 100 nm Whatman Anotop filter. Each vial of 10X SYBR Gold undergoes vortexing, 5 min of sonication bath (BRANSON 200 ultrasonic cleaner), and 5 min of heating at 95 °C. Subsequently, 1.5 mL of the prepared 10x SYBR Gold solution was filtered with a 100 nm filter again and stored it in a 1.5 mL vial wrapped in aluminum foil. Finally, the conditioned media was diluted in 1 × TE buffer to match NTA requirements and mixed with prepared 10X SYBR Gold. The mixture was kept in a rotary mixture for 30 min. For NTA analysis, a negative test was done with only TE buffer and 10X SYBR Gold mix with scatter sensitivity set to 85 and shutter set to 90. For fluorescence analysis, sensitivity at 90 and shutter at 100 was used in NTA. It was made sure the negative test did not show any false positive events. Particle size distribution and concentration were then measured using NTA, and results were interpreted as representing sEVs, in line with MISEV2018 recommendations (https://www.isev.org/assets/Rigor/MISEV2018_Checklist.pdf)^58^.

### ENO1 bioassay

200 nm filtered sEVs were mixed with TRIzol™ LS Reagent (ThermoFisher SCIENTIFIC Inc.) with 250 µL to 750 µL ratio. Solid phase extraction method recommended by the manufacturer (https://assets.thermofisher.com/TFS-Assets/LSG/manuals/trizol_ls_reagent.pdf) was utilized to isolate RNA from the sEV-lysed samples. Isolated RNAs were mixed with functionalized magnetic beads. Streptavidin coated 1 μm magnetic beads (4 mg/mL) were bought from New England Biolabs. The beads were modified with biotinylated oligonucleotides.

A 17 nucleotide long biotinylated ssDNA pulldown sequence- 5′-/5BiotinTEG/AA CGA TGA GAC ACC ATG-3′ was used as pulldowns to isolate ENO1 targets from retrieved RNA mixture. The pulldown was purchased from Integrated DNA Technologies (IDT). The melting temperature of the sequence in 50 mM Na^+^ salt is 49 °C (https://www.idtdna.com/pages/tools/oligoanalyzer). The pulldown sequence targets 1217–1233 region of exon 10 in ENO1 sequence. The E-value for the pulldown sequence was found to be 0.067 which matches 100% of the sequence with human ENO1 (NG_029470.1).

5 µL of the stock 1 μm streptavidin coated New England Bio magnetic beads (4 × 10^9^ beads) were magnetically washed (5 times) and resuspended in 20 nm syringe filtered 1× T50 buffer solution prior to pull down bound magnetic bead preparation. Biotinylated pull down oligos were added to the beads to maintain 1:6 binding site to pull down concentration ratio. The sample-mix was kept in a rotary mixture for 1 h. The unbound excess pulldown sequences were washed off magnetically (3 times). Finally, the pulldown functionalized beads were resuspended for downstream sample preparation at ~ 10^6^/mL concentration.

The sEV derived RNAs were extracted from the initial biofluid of volume 250 µL and resuspended into 50 µL solution. The sample was prepared through sequential heating (95 °C temperature for 5 min first and 1 min for four more times) and annealing (50 °C temperature for 2 min five times) baths. After the first heating bath, 15,000 or 1800 ENO1 pull down functionalized microbeads were mixed with the resuspended RNA sample. After the heating and annealing cycles, the vial containing the RNA and microbead mixture was incubated in an ice bath for 30 min. After incubation, the sample was magnetically washed to discard the unattached RNAs carefully (3 times). Finally, the washed beads were resuspended in 1× T50 with 0.5% (V/V) TWEEN^®^ 20 (Sigma-Aldrich) surfactant for smooth sample delivery to the microfluidic channel.

For the nanopore counting experiment, the inlet reservoir was filled with 6 µL of target functionalized beads and the nanopore reservoir was filled with 6 µL of 1× T50 buffer. The microfluidic channel was filled with 20 kPa negative pressure run through the outlet reservoir for 30–40 s. After that, the outlet reservoir is filled with 6 µL of 1× T50 buffer and 1.5 µL of mineral oil was added on top of the sample in the inlet reservoir. The optical trapping experiment was run for 15 min. After the trapping step, the heater was turned on at 50.5 °C for two and a half minutes.

### Optical and electronic setup

A 532 nm fiber laser (MPB Communications Inc.) was used to trap the microbeads in the protrusion region of the optofluidic chip. The laser was coupled to a single mode fiber through an adjustable fiber-to-fiber coupler (Thorlabs Inc.). Finally, the single-mode fiber was butt coupled to the SC waveguide of the chip. A CCD camera (Andor Luca R, Oxford Instruments) was connected to a custom-built microscope to constitute the bead-monitoring system. A 50X long working distance objective lens (Olympus, SLMPlan, 0.45 NA, 15 mm WD) illuminated and collected light from the device. A 40 nm bandpass filter with 670 nm center wavelength (Omega Optical LLC. 670DF40) was used in the microscope to block the laser light.

A Peltier heater (TES1 12703, Hebei I.T. (Shanghai) Co., Ltd.) was connected to a temperature controller (LDC 3724B, ILX Lightwave) to constitute the heating system for thermodynamic target release from trapped microbeads. The feedback system employed a 10 kΩ thermistor placed on the heater surface and connected to the controller circuit to execute a PID (proportional, integral, and derivative) algorithm.

### Nanopore signal acquisition and translocation analysis

Ag/AgCl electrodes were employed in the nanopore reservoir (trans) and the output reservoir (cis) to apply voltage across the nanopore. The channel is filled with 1× T50 buffer (50 mM NaCl, 10 mM Tris-HCl, filtered with 20 nm filter (Whatman™ Anotop™ 25/0.02)). The nanopore ionic current signal was amplified using a sensitive current amplifier (Axopatch 200B, Molecular Device), and a digitizer (Digidata 1440 A, Molecular Device) was used to record the low pass filtered (cutoff frequency of 10 kHz) signal at a rate of 250 kSa/s.

The recorded current trace was analyzed using a custom MATLAB program to find the translocation events. The algorithm of the MATLAB program relied on the mean and Standard Deviation (SD) check along the current-time trace and a reference trace is created with both information. A user-defined threshold (5× to 7× of the SD) of the baseline ionic current is checked when the reference trace follows through the whole current signal. A five-point group down sampling is done to get the group mean from the current signal in each processing cycle. If the difference between the consecutive five-point means is less than the threshold, the reference mean is updated with a PID algorithm. Otherwise, the program considers the difference as a translocation event and calculates its height. The program further checks if the current returns to the baseline value and if positive, calculates the dwell time. The calculated dwell time is compared with the set threshold time and if it falls beyond that value, the event is not counted as translocation anymore and is discarded as a baseline shift or time-out event.

## Supplementary Information

Below is the link to the electronic supplementary material.

Supplementary Material 1

## Data Availability

All study data are included in the article and the supplementary information. No new RNA sequence data were generated in this study. For NCBI BLAST matching, ENO1 sequence with GenBank accession number NG_029470.1 is used which is publicly available at https://www.ncbi.nlm.nih.gov/nuccore/NG_029470.1.
