# Supplementary material for: Single molecule nanopore counting assay targeting small extracellular vesicle cargo for non-invasive monitoring of cerebral organoid development and health
Source: Sci Rep. 2025 Dec 12;15:45806. doi: 10.1038/s41598-025-31284-8 (PMC12756300; doi:10.1038/s41598-025-31284-8)
Supplement: Supplementary file 1 — Supplementary Material 1 [file 41598_2025_31284_MOESM1_ESM.docx]

**Supplementary Information of**

Single molecule nanopore counting assay targeting small extracellular vesicle cargo for non-invasive monitoring of cerebral organoid development and health

S M Saiduzzaman^a^, Ruiting Xu^a^, Mohammad Julker Neyen Sampad^a^, Ryan N. Hoffman^b^, Spencer T. Seiler^c^, Quinton Brail^b^, Viktor Yurevych^b^, Zachary J. Walker^d^, Tanner N. Wells^d^, Ephraim M. Ong^d^, Thomas D. Yuzvinsky^a^, Aaron R. Hawkins^d^, Sofie R. Salama^b^, Mircea Teodorescu^a^, David Haussler^c^, and Holger Schmidt*^a^

**^a^School of Engineering, University of California, Santa Cruz, CA 95064, USA**

**^b^Department of Molecular, Cell, & Developmental Biology, University of California, Santa Cruz, CA, USA**

**^c^Department of Biomolecular Engineering, University of California, Santa Cruz, CA, USA**

**^d^Electrical and Computer Engineering Department, Brigham Young University, Provo, UT 84602, USA**

**Corresponding Author:** Correspondence to Holger Schmidt (hschmidt@soe.ucsc.edu)

**Metaflex monitoring of Organoid glucose consumption:**

The goal of the manuscript was to monitor a glycolytic stress monitoring marker ENO1 non-invasively (EV derived) from organoids grown and maintained in different glucose conditions. The glucose concentrations in the organoid media were monitored and characterized every 48 hours after media exchange. Figure S1 shows the glucose monitoring using metalfex glucose calculator depicting the initial concentration at 0^th^ hour and the 48^th^ hour and the concentration drop due to glucose consumption by organoids for both cohort of glucose-controlled media. 200uL of the conditioned media was used for metalfex measurements.

| 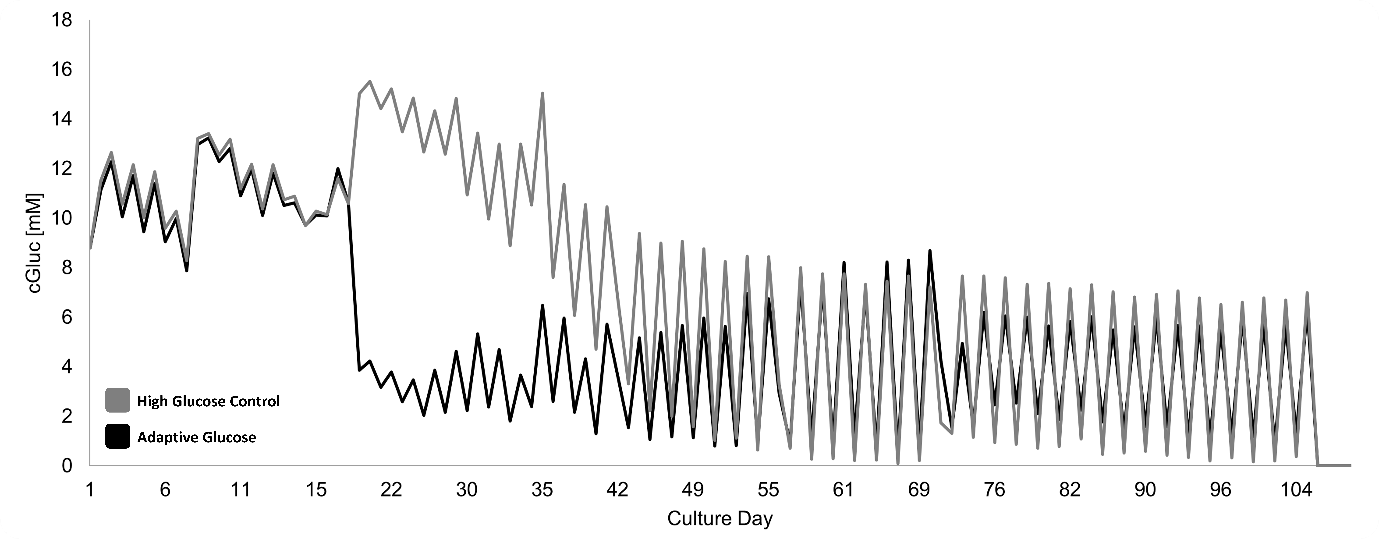 |
| --- |
| Figure S1- Glucose level in high and adaptive glucose media measured by metaflex calculator |

**Immunohistochemistry characterization of organoids**

The organoids were also characterized by immunohistochemistry for standard organoid markers along their maturation periods. Figure S2 shows organoid markers PAX6 (green) and CTIP2 (magenta) for Day 70 organoids grown in both adaptive and high glucose media.

| 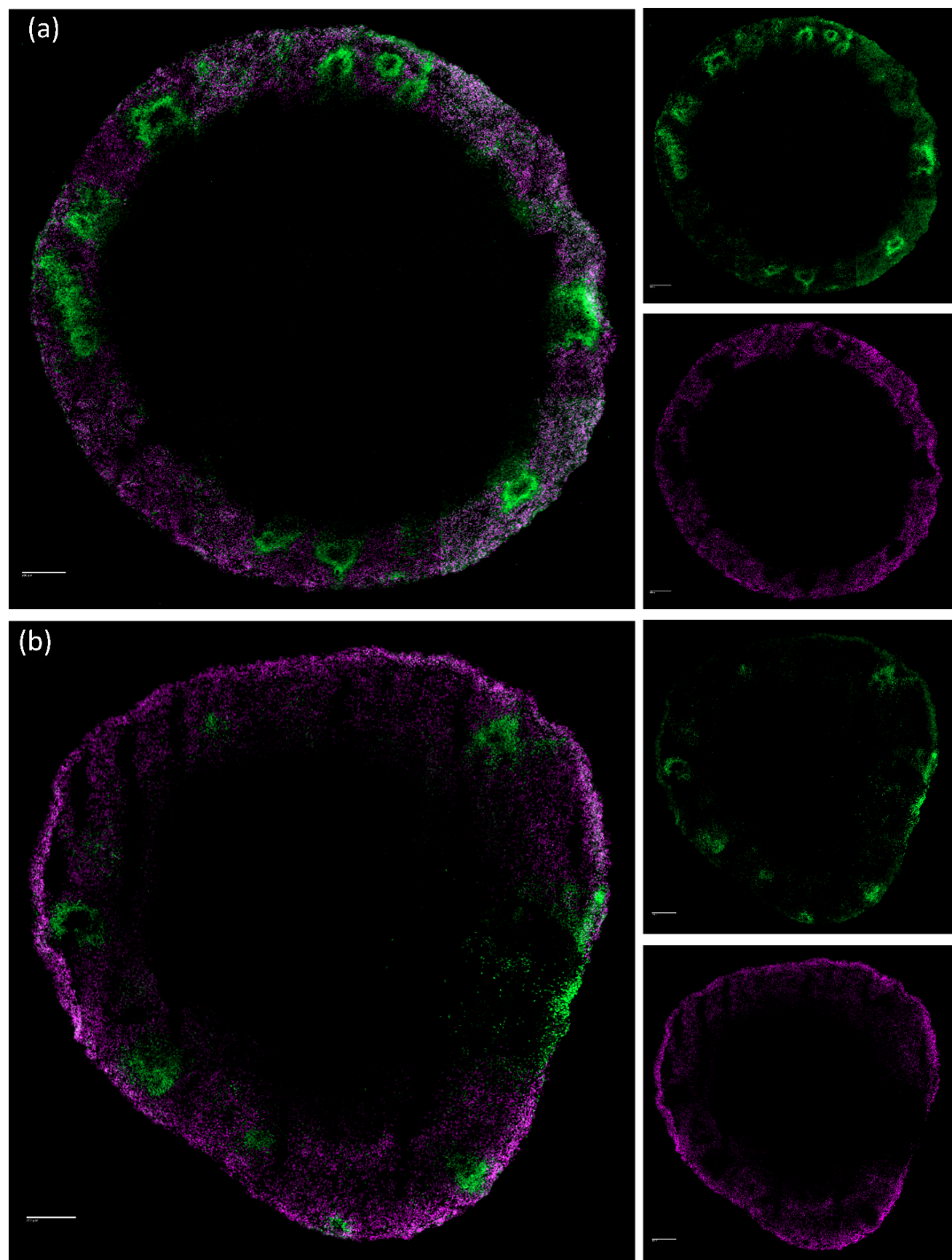 |
| --- |
| Figure S2- Immunohistological analysis of serial sections from whole organoid samples from High Glucose culturing and Adaptive Glucose using cerebral specified cell markers. Shown in green, PAX6 is a cerebral specified radial glia marker. Shown in magenta, CTIP2 (also BCL11B) is a marker for cerebral specified deep layer neurons, marking both layer V and layer VI excitatory neuron subtypes. **A**. Immunohistological images of representative organoids from the High Glucose condition showing the development of neural rosettes at day 70. **B.** Immunohistological images of representative organoids from the Adaptive Glucose condition showing some development of neural rosettes at day 70. Scale bars are equal to 200µm. |

**Negative control current trace for Optofluidic nanopore sensor:**

The EV ENO1 Nanopore assay has been validated for false positives prior to detecting cell-line derived EV ENO1 with this assay. Target negative microbeads were prepared with oligo pulldown, run through the nanopore chips, and trapped with optical beam. The stage was heated at 50C and nanopore currents were monitored. No false negative signals were detected during a 5-minute trace with the heater on. Figure S3 contains a negative current trace snippet from the experiment.

|  |
| --- |
| Figure S3- Nanopore current trace after heating at 50C where beads functionalized with oligo-pull down (without target attachment) are used. |

**ENO1 assay characterization in optofluidic nanopore sensor with ssDNA:**

The ENO1 nanopore assay was validated with synthetic ssDNA matching ENO1 sequence for TACRE measurements in optofluidic nanopore sensor. The ssDNA ENO1 was spiked with TrizolLS lysis buffer and pulldown functionalized beads were used to capture the ENO1 ssDNA. Then, the beads were magnetically washed and resuspended in the nanopore salt buffer (1XT50, pH=6.92). Finally, the beads were run through and trapped in the optofluidic nanopore sensor and ssDNA targets were captured and counted by the nanopore after thermal release as shown in Figure S4. The significant match between concentration retrieved from the nanopore sensor and the expected concentration establishes the validity of the pulldown assay.

| 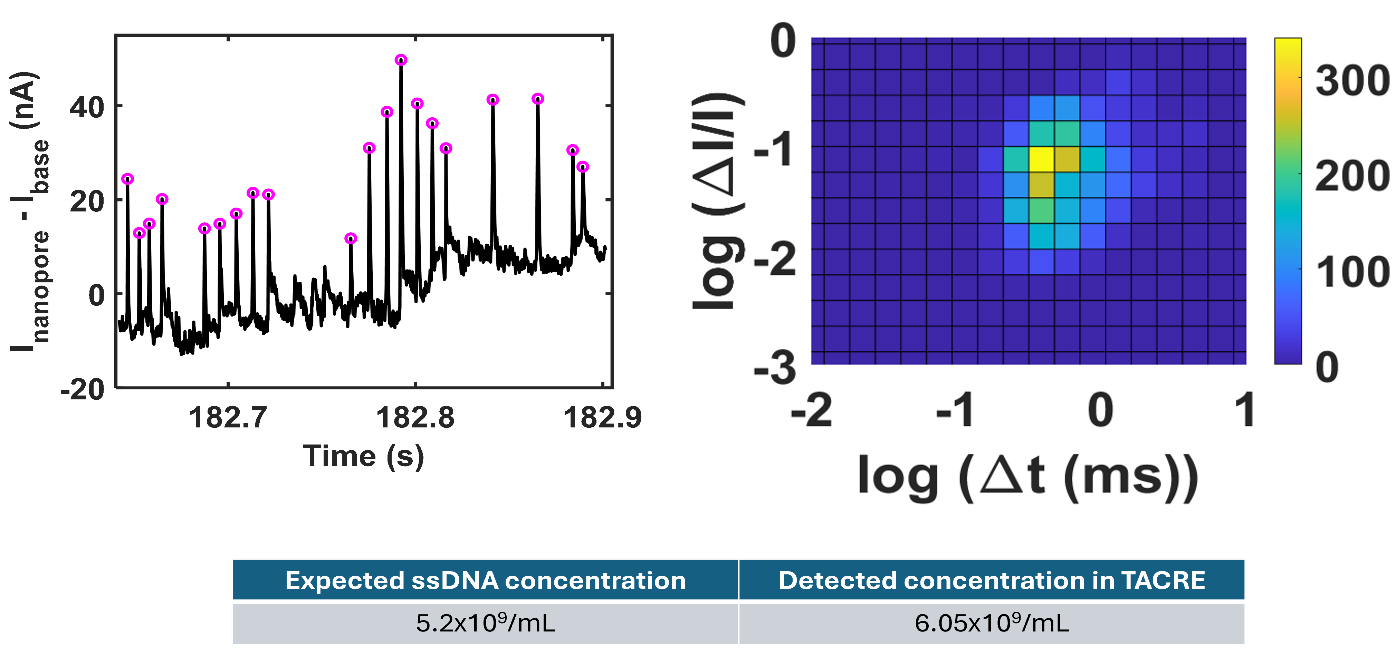 |
| --- |
| Figure S4: ENO1 ssDNA were spiked with lysis buffer and captured with beads+pull down assembly. |

**Optofluidic nanopore counting parameters for ENO1 quantification from 0.5 mL sample volume**

Following Table enlists the bioassay parameter – total number of beads ($n_{beads}$) used to capture ENO1 mRNA from 0.5mL sample volume and the nanopore experimental parameters- number of beads trapped in chip ($n_{trapped}$) and number of ENO1 mRNA events ($n_{events}$) detected by the nanopore after thermal release. These parameters are sufficient for determining the concentration of the EV derived ENO1 mRNA $c_{mRNA}$. Relative Standard Deviation (RSD) estimates the binomial distribution-based uncertainty for the measurements, which is a function of the parameters mentioned above (from equation 1 in the manuscript).

**Table S1**

| **Days Media** | **30** | **49** | **67** | **72** | **82** | **102** |
| --- | --- | --- | --- | --- | --- | --- |
| **Adaptive**  **glucose** | $n_{trapped}=10$  $n_{events}=458$  $n_{beads}=15000$  $c_{mRNA}=1.4\times{10}^{6} mL^{-1}$  $RSD=4.63 \%$ | $n_{trapped}=13$  $n_{events}=1097$  $n_{beads}=18000$  $c_{mRNA}=3.15 \times{10}^{6} mL^{-1}$  $RSD=2.96\%$ | $n_{trapped}=4$  $n_{events}=513$  $n_{beads}=18000$  $c_{mRNA}=4.7\times{10}^{6} mL^{-1}$  $RSD=4.38 \%$ | $n_{trapped}=4$  $n_{events}=688$  $n_{beads}=15000$  $c_{mRNA}=5.34 \times{10}^{6} mL^{-1}$  $RSD=3.75 \%$ | $n_{trapped}=6$  $n_{events}=752$  $n_{beads}=15000$  $c_{mRNA}=3.92 \times{10}^{6} mL^{-1}$  $RSD=3.57 \%$ | $n_{trapped}=7$  $n_{events}=81$  $n_{beads}=15000$  $c_{mRNA}=3.6 \times{10}^{5} mL^{-1}$  $RSD=10.91 \%$ |
| **High glucose** | $n_{trapped}=13$  $n_{events}=568$  $n_{beads}=15000$  $c_{mRNA}=1.36 \times{10}^{6} mL^{-1}$  $RSD=4.12 \%$ | $n_{trapped}=4$  $n_{events}=186$  $n_{beads}=15000$  $c_{mRNA}=1.4 \times{10}^{6} mL^{-1}$  $RSD=7.32 \%$ | $n_{trapped}=27$  $n_{events}=468$  $n_{beads}=18000$  $c_{mRNA}=6.4 \times{10}^{5} mL^{-1}$  $RSD=4.56 \%$ | $n_{trapped}=6$  $n_{events}=380$  $n_{beads}=15000$  $c_{mRNA}=1.9 \times{10}^{6} mL^{-1}$  $RSD=5.13 \%$ | $n_{trapped}=9$  $n_{events}=413$  $n_{beads}=15000$  $c_{mRNA}=1.38 \times{10}^{6} mL^{-1}$  $RSD=4.91 \%$ | $n_{trapped}=3$  $n_{events}=188$  $n_{beads}=15000$  $c_{mRNA}=2.32 \times{10}^{6} mL^{-1}$  $RSD=6.56 \%$ |
